# Reduced Pancreatic Exocrine Function and Organellar Disarray in a Canine Model of Acute Pancreatitis

**DOI:** 10.1371/journal.pone.0148458

**Published:** 2016-02-19

**Authors:** Yuepeng Jin, Yongyu Bai, Qiang Li, Pravin Avinash Bhugul, Xince Huang, Lewei Liu, Liangliang Pan, Haizhen Ni, Bicheng Chen, Hongwei Sun, Qiyu Zhang, Michael Hehir, Mengtao Zhou

**Affiliations:** 1 Department of Surgery, The First Affiliated Hospital, Wenzhou Medical University, Wenzhou, Zhejiang Province, China; 2 Wenzhou Medical University, Wenzhou, Zhejiang Province, China; 3 YueQing Affiliated Hospital of Wenzhou Medical University, YueQing People’s Hospital, Yueqing, Zhejiang Province, China; 4 Zhejiang Provincial Top Key Discipline in surgery, Wenzhou Key Laboratory of Surgery, Department of Surgery, The First Affiliated Hospital, Wenzhou Medical University, Wenzhou, Zhejiang Province, China; 5 Ningbo University Medical School, Ningbo, Zhejiang Province, China; University of Szeged, HUNGARY

## Abstract

The aim of the present study was to investigate the pancreatic exocrine function in a canine model and to analyze the changes in organelles of pancreatic acinar cells during the early stage of acute pancreatitis (AP). AP was induced by retrograde injection of 5% sodium taurocholate (0.5 ml/kg) into the main pancreatic duct of dogs. The induction of AP resulted in serum hyperamylasemia and a marked reduction of amylase activity in the pancreatic fluid (PF). The pancreatic exocrine function was markedly decreased in subjects with AP compared with the control group. After the induction of AP, histological examination showed acinar cell edema, cytoplasmic vacuolization, fibroblasts infiltration, and inflammatory cell infiltration in the interstitium. Electron micrographs after the induction of AP revealed that most of the rough endoplasmic reticulum (RER) were dilated and that some of the ribosomes were no longer located on the RER. The mitochondria were swollen, with shortened and broken cristae. The present study demonstrated, in a canine model, a reduced volume of PF secretion with decreased enzyme secretion during the early stage of AP. Injury of mitochondria and dilatation and degranulation of RER may be responsible for the reduced exocrine function in AP. Furthermore, the present model and results may be useful for researching novel therapeutic measures in AP.

## Introduction

Acute pancreatitis (AP) is a common, often life-threatening, inflammatory disease of the exocrine pancreas and is associated with a significant morbidity and mortality [1,2]. However, its pathogenesis remains obscure. Multiple factors are involved in the initiation and development of AP, making it difficult to choose specific or effective treatments for the prevention of complications. A common feature in AP is the intra-acinar cell activation of zymogens, thus leading to autodigestion of the gland [3,4]. In the past decade, the focus of research has been on signaling pathways involved in the inflammatory cell infiltrate and the systemic inflammatory response [4–9].

In contrast with the recent research, very little is known regarding the pancreatic exocrine function in AP. A further gap in knowledge is associated with electron microscopy findings in AP, particularly those related to dysfunction and disarray of acinar cell organelles, such as lysosomes, ribosomes, endoplasmic reticulum, and mitochondria.

During the development of AP, both acinar and ductal cells of the exocrine pancreas are damaged [10]. This damage can affect the secretory processes, which are important for digestion, and also threaten the integrity of the pancreas.

The aim of this study was to observe pancreatic exocrine function during experimental AP in dogs and to gain insight into the ultrastructural changes in the pancreatic cells.

## Materials and Methods

### Animals and Groups

The Laboratory Animal Center of Wenzhou Medical University (Wenzhou, China) supplied twelve adult mixed-breed dogs of both sexes (weighing between 10 and 14 kg). The dogs were fed water and standard dog food (ke Ao Xie li Co. Ltd, Beijing, China). The dogs were maintained at a temperature of 20–26°C and with a 12-h light-dark cycle. The animals were randomly divided into 2 groups: the control group (n = 6) and the AP group (n = 6). AP was induced by the retrograde intraductal infusion of 5% sodium taurocholate.

### Ethics Statement

The Institutional Animal Committee of Wenzhou Medical University, Wenzhou, China, approved the protocol for the animal experiment (Permit Number: wydw2011-0001). All animals received care in accordance with the ‘Guide for the Care and Use of Laboratory Animals’. The animals were monitored throughout the experiment. During the experimental periods, canines were administered intravenous total parenteral nutrition. The dogs were maintained in good condition and showed no signs of weight loss. If there were signs of pain or distress, we performed an intramuscular injection of dolantin (1 mg/kg). At the end of the experiment, the animals were euthanized using an overdose of 1.5% pentobarbital sodium (4–5 ml/kg).

### The Animal Model

The dogs were not fed with food for 12 h and with water for 4 h before the operation, and they were anesthetized by an intramuscular injection of 5% hydrochloric acid ketamine (1 ml/5 kg) and an intravenous injection of 1.5% pentobarbital sodium (1.5 ml/kg, Beijing Chemical Reagent Company, Beijing, China). Half an hour before the operation, 0.5% metronidazole (50 ml) and cefotaxime sodium (0.5 g) were injected as infection prophylaxis. After an upper abdominal midline incision, the wall of the descending duodenum was incised and cannulated using a central venous catheter through the ampulla of Vater into the main pancreatic duct. The catheter was then fixed on the duodenal wall and the skin. Twenty-four hours after the establishment of the model, AP was induced by retrograde injection of 5% sodium taurocholate (0.5 ml/kg; Sigma Chemical Company, St. Louis, MO, USA) via the central venous catheter through a microinjection pump at a speed of 1 ml/min under anesthesia. The control group underwent the same surgical procedure and the same cannulation but without infusion. During the recovery and experimental periods, the dogs were conscious, were not fed orally and were all administered continuous total parenteral nutrition (TPN) through infusion via the right cephalic vein. The nutritional admixture contained protein, carbohydrates, and electrolytes, as shown in Table 1. Using amino acid 8.5% [compound amino acid injection], (sino-swed pharmaceutical Co. Ltd), the admixture was prepared and transferred into a sterile plastic infusion bag under aseptic conditions. The resulting regimen provided approximately 50 kcal/kg, 0.25 g nitrogen/kg, Na^+^ 2 mol/kg, and K^+^ 1 mol/kg. It was suitable as a low-osmolarity parenteral nutrition admixture for administration via the peripheral venous circulation. The fluid intake was 100–120 ml/kg body weight per day. Pure PF was collected via the central venous catheter that was cannulated into the main pancreatic duct in 24-h aliquots, and it was not injected back into the duodenum. Sample collection began after 1 h of stabilization and was then performed every 24 h thereafter.

**Table 1:** Composition of TPN Formulation.

Admixture Component	Amount (ml) in TPN
Amino acids	250
50% glucose solution	441
Sterile water for injection	784
10% Sodium chloride	15
10% Potassium chloride	10

### Collection of Specimens and Assays

1. The following assays were performed on the collected 24-hr aliquots of PF from each subject: amylase, lipase, protein, bicarbonate concentration, and pH. The bicarbonate concentration and pH value were determined by an AVL Compact 3 blood gas analyzer (AVL medical instrument company, Switzerland). Amylase activity, lipase activity, and protein concentration in the PF were determined by the Hitachi 7600 automatic biochemical analyzer (Hitachi, Ltd., Tokyo, Japan).
2. The PF volume was determined using a calibrated measuring cylinder.
3. Blood was collected via the left cephalic vein preoperatively and at 1, 12, 24, and 48 h after induction of AP. Serum was stored at -80°C until further testing. Serum amylase activity was determined by iodine-amylum colorimetry, and serum lipase activity was assessed by enzymatic colorimetry; both are expressed in U/L.
4. Specimens of the head of the pancreas, about 1.5 cm from the main pancreatic duct, were harvested after the dogs were euthanized on day 3 (between 72 and 73 h after induction of AP) for histopathological and electron microscopy examination.

### Histopathological Analysis

A portion of the head of the pancreas was harvested and fixed in 10% formaldehyde solution, embedded in paraffin, sectioned, and stained with hematoxylin-eosin (H&E) for light microscopy.

### Transmission Electron Microscopy

For transmission electron microscopy assessment, 1-mm^3^ pieces of the pancreas from each dog were fixed in 2.5% phosphate-buffered glutaraldehyde for one week. The samples were washed with 0.1% sodium phosphate buffer before being post-fixed in 1% osmium tetroxide for 2 h at 4°C, and then washed with 0.1% sodium phosphate buffer again. After dehydration in a graded series of ethanol, the samples were embedded in Epon-812. Ultrathin sections were double-stained with uranyl acetate and lead citrate and examined with a transmission electron microscope (Hitachi, Ltd., Tokyo, Japan).

### Statistical Analysis

The Statistical Package for the Social Sciences software SPSS version 18.0 (SPSS, Inc., Chicago, IL, USA) was used to compute the means ± standard deviations (SD). Parameters demonstrating equal variances and normal distributions were analyzed by Student’s t test (lipase activity, bicarbonate concentration, and pH value in PF). Parameters demonstrating unequal variances were analyzed by the Mann-Whitney U test (PF volume, amylase, and protein concentration in PF). The serum amylase and lipase activities were analyzed by two-way analysis of variance (ANOVA) followed by the Games-Howell test as a post-hoc test. For all analyses, p < 0.05 was considered to indicate significant differences.

## Results

### PF Volume, Exocrine Amylase, Lipase, and Protein Concentration in PF during Experimental AP

**Fig 1:**
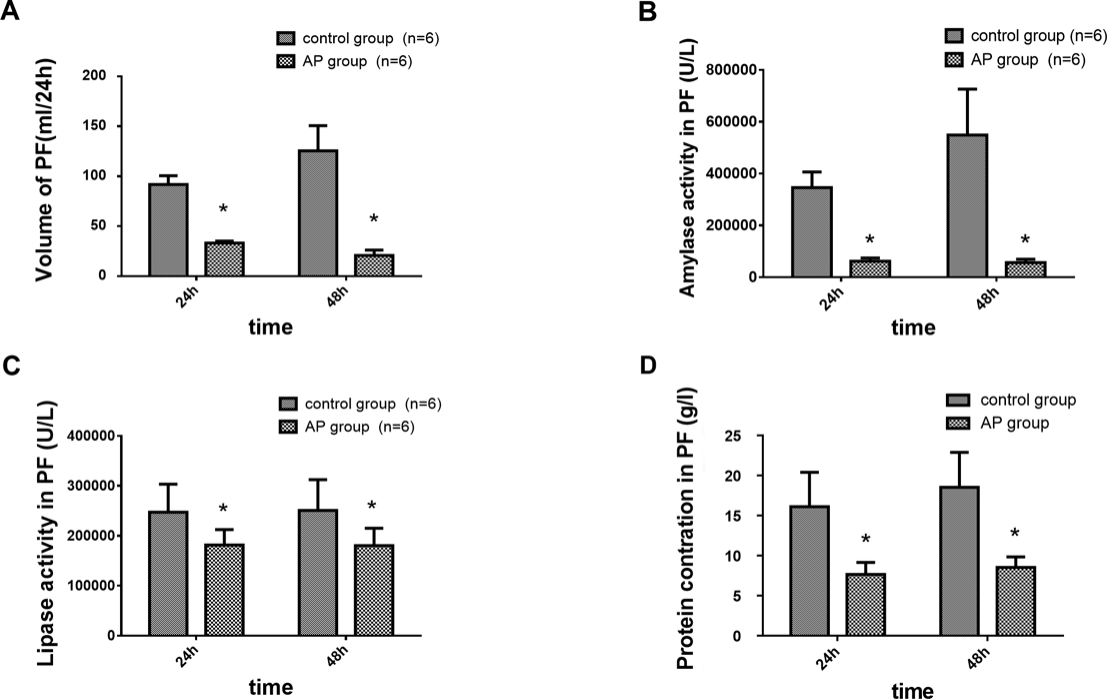
The PF volume, outputs of amylase activity, lipase activity, and protein concentration in the PF in a canine AP model. AP was induced by a retrograde injection of 5% sodium taurocholate (0.5 ml/kg) via the main pancreatic duct. (A): volume of PF (ml/24 h), (B): amylase activity in PF (U/l), (C): lipase activity in PF (U/l), (D): protein concentration in PF (g/l), (n = 6 dogs per group). The PF volume, amylase activity, lipase activity, and protein concentration in the PF were significantly decreased after the induction of AP compared with the control group. *P < 0.05, the AP group vs. the control group at both time points.

As shown in Fig A in S1 Data, the PF volume showed a decrease compared with that from the control group on days 1 and 2 after the induction of AP. Exocrine outputs of amylase and lipase in the PF were significantly decreased after the induction of AP and remained decreased during the experimental period (Figs B, C in S1 Data). The protein concentration in the PF, shown in Fig D in S1 Data, markedly decreased after the induction of AP and gradually increased but was significantly lower than that in the control group. These results indicated that the basal PF secretion and enzyme secretion were reduced during the early stage of AP (Fig 1).

### Bicarbonate Concentration and pH Value in the PF during Experimental AP

**Fig 2:**
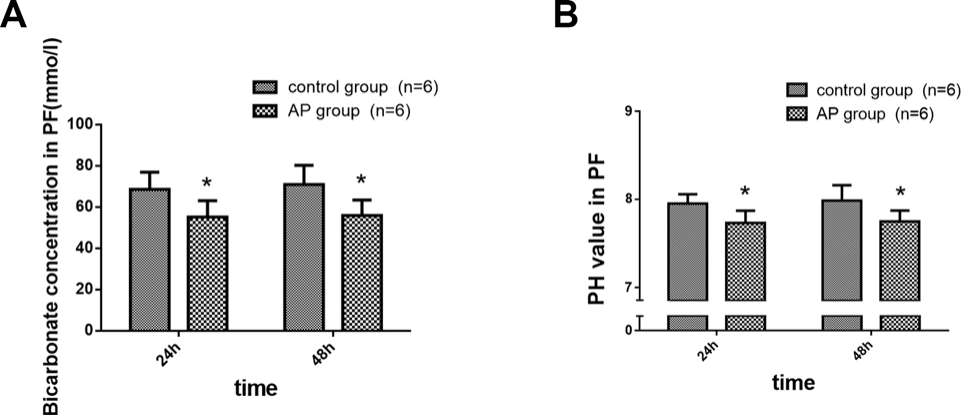
Bicarbonate concentration and pH value in the PF in a canine AP model. AP was induced by a retrograde injection of 5% sodium taurocholate (0.5 ml/kg) via the main pancreatic duct. (A) Bicarbonate concentration in the PF, (B) pH value of the PF, (n = 6 dogs per group). The bicarbonate concentration and pH value in the PF were significantly decreased after AP induction compared with the control group. *P < 0.05, the AP group vs. the control group at both time points.

The bicarbonate concentration and pH value in the PF were significantly decreased in the experimental AP subjects compared with the control group (Fig 2) (Fig E in S1 Data).

### Serum Amylase and Lipase Activities

**Fig 3:**
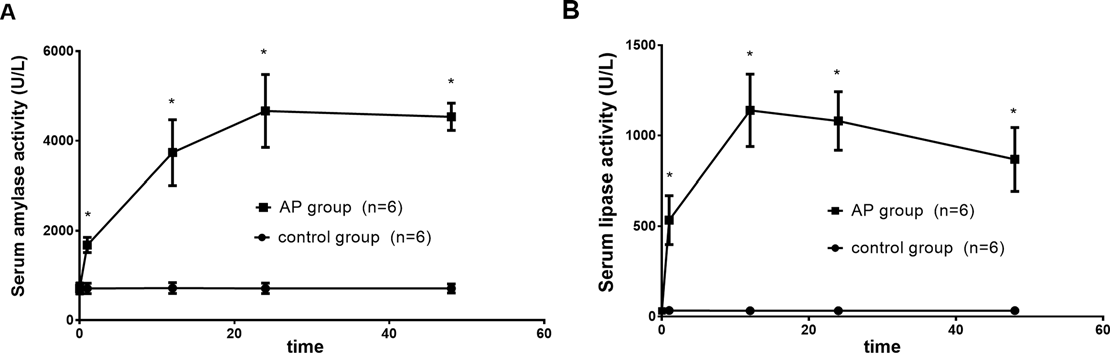
Serum amylase and lipase activities (U/L). Time course of activities of serum amylase and lipase before the operation (0 h) and 1, 12, 24, and 48 h after the administration of 5% sodium taurocholate. (A) Serum amylase activity (U/l) and (B) serum lipase activity (U/l), (n = 6 dogs per group). The serum amylase and lipase activities were significantly increased following the induction of AP compared with the control group. *P < 0.05, the AP group vs. the control group at each time point.

The serum amylase and lipase activities were significantly increased following the induction of AP; the serum amylase reached a peak at 24 h, whereas the lipase peaked at 12 h (Fig F in S1 Data). The serum amylase and lipase activities did not return to baseline values until 48 h after the induction of AP (Fig 3).

### Histopathological Examination

**Fig 4:**
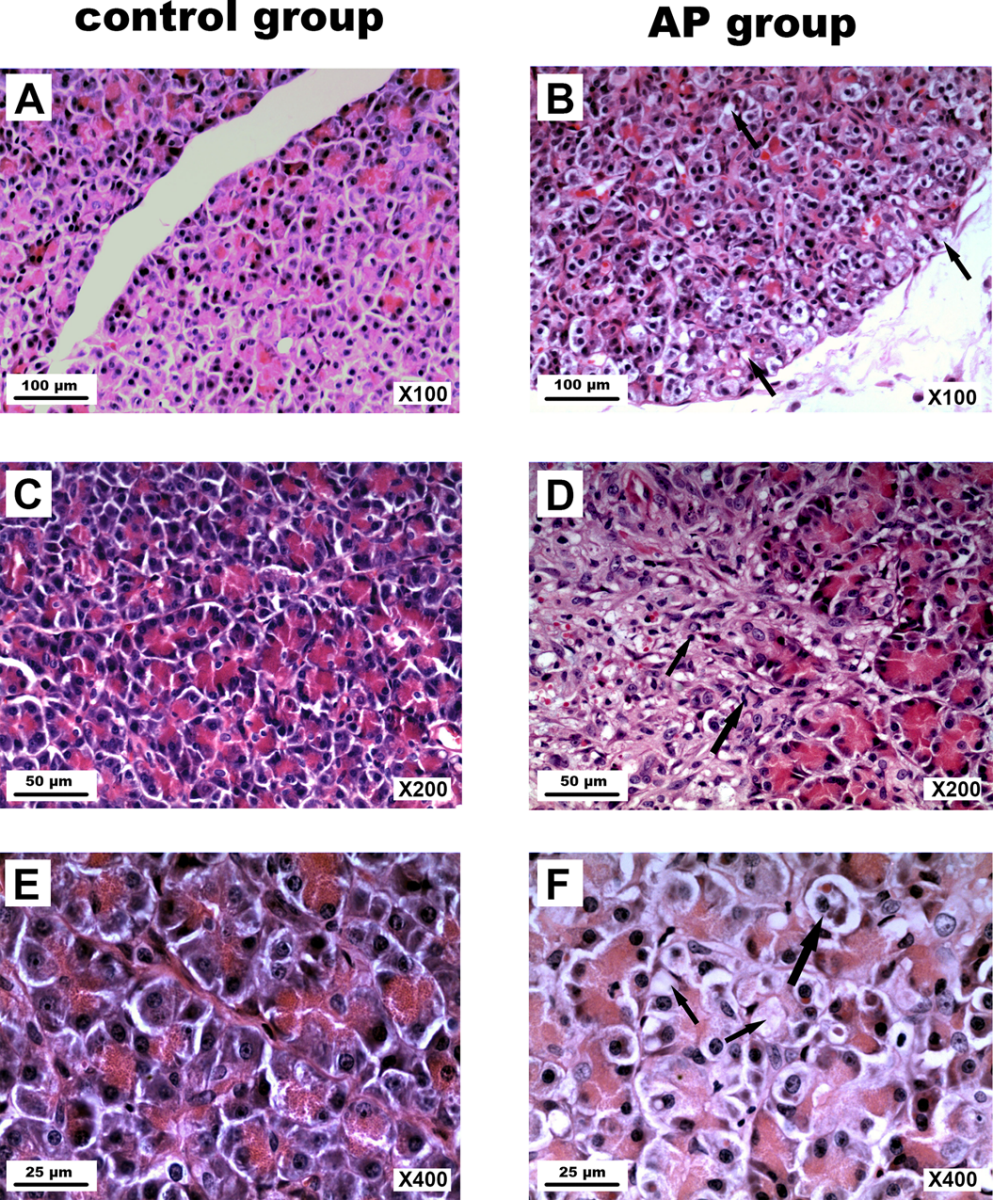
Histopathological examination of the canine pancreas. (A) A pancreas from the control group (scale bar = 100 μm). (B) A pancreas from the AP group (scale bar = 100 μm). (C) A pancreas from the control group (scale bar = 50 μm). (D) A pancreas from the AP group (scale bar = 50 μm). (E) A pancreas from the control group (scale bar = 25 μm). (F) A pancreas from the AP group (scale bar = 25 μm). The pancreas from the control group exhibited normal lobular architecture (A, C, D). The pancreas from the AP group showed features of mild AP with acinar cell edema (F, large arrow), cytoplasmic vacuolization (F, small arrow; B, arrow), fibroblasts infiltration (D, large arrow), and inflammatory cell infiltration (D, small arrow) in the interstitium.

Histopathological examination of the canine pancreas from the control group and the AP group showed in Fig 4. The control group without sodium taurocholate showed no morphological evidence of pancreatic injury (Fig A, B, C in S1 Fig). Histopathologically, H&E staining of pancreatic tissues derived from sodium taurocholate-induced AP demonstrated that dogs developed mild AP, including acinar cell edema, cytoplasmic vacuolization, fibroblasts infiltration, and inflammatory cell infiltration in the interstitium (Fig D, E, F in S1 Fig).

### Electron Microscopy Results

**Fig 5:**
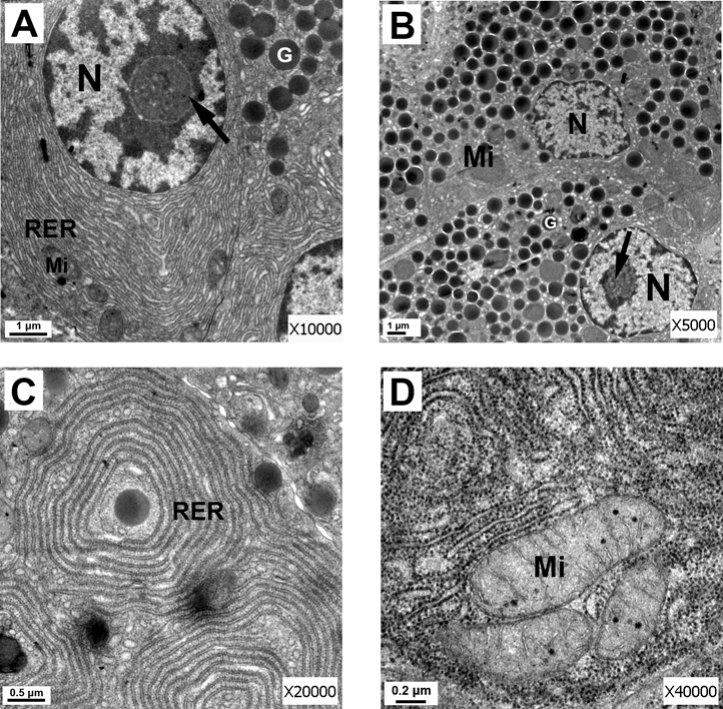
Electron micrographs of the canine pancreas from the control group. (A) Electron micrograph of pancreatic acinar cells from the control group, demonstrating normal acinar cellular architecture. Cell nucleus (N), nucleolus (arrow), zymogen granule (G), mitochondria (Mi) with recognizable cristae, and rough endoplasmic reticulum (RER) with attached ribosomes are shown (scale bar = 1 μm). (B) Electron micrograph of normal pancreatic acinar cells from the control group. Cell nucleus (N), nucleolus (arrow), zymogen granule (G), mitochondria (Mi), and rough endoplasmic reticulum (RER) are shown. There were many mature zymogen granules oriented toward the apical cell membrane (scale bar = 2 μm). (C) Electron micrograph of rough endoplasmic reticulum (RER) from the control group. The RER did not show dilatation, and numerous ribosomes were attached (scale bar = 0.5 μm). (D) Electron micrograph of mitochondria (Mi) from the control group. The mitochondria with recognizable cristae (scale bar = 0.2 μm).

Electron microscopy was performed on thin sections of the head of the pancreas. Electron micrographs of the canine pancreas from the control group showed in Fig 5. Fig A and Fig B in S2 Fig show the appearance of normal pancreatic acinar cells, which were pear-shaped with round nuclei located in the base, and each nucleus had 1 or 2 nucleoli. Obvious chromatin could be seen under the nuclear membrane. Golgi complexes were well developed, and there were many mature zymogen granules oriented toward the apical cell membrane (Fig B in S2 Fig). The cytoplasm in the basal location was filled with rough endoplasmic reticulum (RER) with ribosomes attached to the surface (Fig C in S2 Fig). The mitochondria were observed to have recognizable cristae (Fig D in S2 Fig).

**Fig 6:**
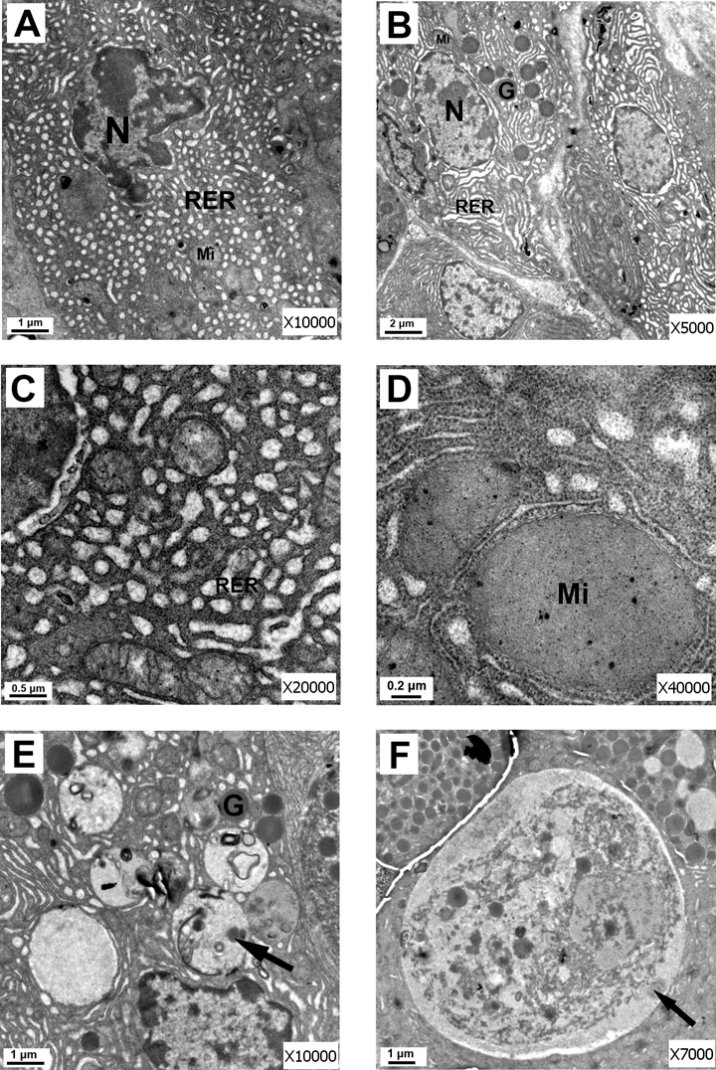
Electron micrographs of the canine pancreas from the AP group. (A) Electron micrograph of pancreatic acinar cells from the AP group showing generalized disorganization. Mild pyknosis of the nucleus (N), mitochondria (Mi), and rough endoplasmic reticulum (RER) are shown. Mitochondria exhibited a swollen appearance with short and broken cristae. The RER showed a loss of the normal pattern (compared with Fig 5A) with dilated irregular sacs, and some of the ribosomes were no longer located on the RER (scale bar = 1 μm). (B) Electron micrograph of pancreatic acinar cells from the AP group. Mild pyknosis of the nucleus (N), mitochondria (Mi), and rough endoplasmic reticulum (RER) are shown. The amount of zymogen granules in the apical zone was reduced compared with Fig 5B (scale bar = 2 μm). (C) Electron micrograph of RER from the sodium taurocholate-induced AP group. The RER showed extensive dilatation, and some of the ribosomes were no longer located on the RER (scale bar = 0.5 μm). (D) Electron micrograph of mitochondria (Mi) from the sodium taurocholate-induced AP group. The mitochondria were swollen and contained short and broken cristae (scale bar = 0.2 μm). (E) Electron micrograph of autophagic vacuoles from the AP group. Large autophagic vacuoles were seen (arrow) (scale bar = 1 μm). (F) Electron micrograph of acinar cell necrosis (arrow) from the AP group (scale bar = 1 μm).

Electron micrographs of the canine pancreas from the AP group showed in Fig 6. Fig A and Fig B in S3 Fig illustrate a representative characteristic feature of AP in a dog in the AP group. The pancreatic acinar cells showed mild pyknosis of the nucleus. The amount of zymogen granules in the apical zone was significantly reduced; interstitial collagen fibers appeared proliferated; and cells were smaller in size (Fig A, B in S3 Fig). Most of the RER were dilated, and some of the ribosomes were no longer located on the RER (Fig C in S3 Fig). The mitochondria were swollen and contained short and broken cristae (Fig D in S3 Fig). Large autophagic vacuoles were seen (Fig E in S3 Fig). Individual acinar cells showed necrosis (Fig F in S3 Fig).

## Discussion

The present study showed that pancreatic exocrine function was decreased in the canine model of AP, which was induced by the injection of 5% sodium taurocholate (0.5 ml/kg) via the pancreatic duct cannula. PF volume and exocrine amylase, lipase, protein, bicarbonate, and pH value were all decreased in the AP dogs. AP was manifested by serum hyperamylasemia, acinar cell edema, cytoplasmic vacuolization, fibroblasts infiltration, and inflammatory cell infiltration in the interstitium. This model reflected mild pancreatitis that did not reach the level of severe autodigestion of the gland. Because our specific aim was to determine how experimental AP alters pancreatic exocrine function, we used a conscious canine AP model in which the dogs had completely recovered from the abdominal operation and pancreatic duct cannulation to enable testing of optimal pancreatic exocrine function.

Pancreatic exocrine function during the early stage of AP is still controversial. Czako et al. reported that protein output in PF was reduced in pancreatitis models induced by cerulein or sodium taurocholate, but the basal PF secretion was considerably elevated at the initiation of pancreatitis [11,12]. Czako et al. used a rat model in which the pylorus was ligated and the bile and pancreatic ducts were cannulated [11,12]. The detailed procedure of the model is as follows: the bile duct was ligated proximal to the pancreas below the hilum of the liver, and a tube was inserted into the bile duct above the ligature; a pancreatic cannula was then inserted into the distal end of the common bile-pancreatic duct at its entrance to the duodenum and tied in place [11,12]. However, Murayamma et al. developed a conscious rat experimental model with gastric, duodenal, bile, and pancreatic fistulas and found that basal and cholecystokinin-stimulated pancreatic juice flow and protein secretion were significantly decreased after cerulein-induced AP [13]. The procedure used in their study is as follows: the duodenal loop was identified, and a catheter was inserted into the common bile-pancreatic duct proximal to the ampulla of Vater; the bile duct was ligated proximal to its entry into the pancreas; another catheter was placed into the bile duct between the ligature and the confluence of the hepatic ducts to drain bile; a fourth catheter was inserted into the duodenum 1 cm distal to the pylorus; and a gastric fistula was created in the mid-stomach and was brought through the abdomen [13]. Niederau et al. evaluated pancreatic secretion in rat and mouse models. In the rat model, the pancreaticobiliary duct was cannulated through the duodenal papilla with polyethylene tubing [14]. In this case, the samples, namely, the PF and bile, were collected from the tube. For the mouse model, because of the small size of mice, cannulation of the pancreatic duct was not possible. A 5-cm duodenal loop was made to collect the duodenal fluid [14]. The amount of enzyme in the duodenal fluid was determined, and the samples, including the pancreatic fluid, bile, and duodenal juice, were collected from the duodenal loop. Niederau et al. found a secretory blockade during pancreatitis, and the exocrine pancreas gradually became resistant to cholecystokinin stimulation after the onset of AP [14]. Therefore, it seemed to be important to clarify the pancreatic exocrine function during the early stage of experimental AP. Our findings were consistent with those from the study by Murayamma et al. [13], in which the pancreatic basal secretion was reduced during the early stage of AP. However, our finding of PF secretion was inconsistent with the findings of the studies by Czako et al. and Sata et al., who reported that the basal PF secretion was elevated after the induction of AP [11,12,15]. This discrepancy in results between the present study and the other studies cited above may be attributed to the differences in the experimental design.

In our canine model, the pancreatic duct was cannulated in the duodenal wall with a central venous catheter. The catheter was guided extracorporeally and fixed on the abdominal skin. Twenty-four hours after the establishment of the model, AP was induced by retrograde injection of 5% sodium taurocholate (0.5 ml/kg) via the central venous catheter under anesthesia. The major differences between the present study and the other cited experiments are the following. (1) Different species: our experiment used dogs, whereas the above experiments used a rat model or mouse model. (2) Different model establishment: our model required pancreatic duct cannulation for the collection of pure PF, whereas the above models were much more complicated and different from each other. The duodenal loop in the mouse model was used to collect the PF, bile, and duodenal juice. The different rat models were mentioned in the previous chapter. (3) Different methods of inducing AP: AP in our study was induced by 5% sodium taurocholate (0.5 ml/kg), whereas that in the above models was induced by sodium taurocholate, cerulein, or feeding the animals an ethionine-supplemented, choline-deficient diet. (4) Different time points for PF collection: PF was collected every 24 h in our study, whereas that in the above studies was collected every 30 min or 1 h. (5) Different states of consciousness: our experiment employed a conscious canine AP model to collect PF, whereas the above experiments were performed under anesthesia, except for the experiment performed by Murayamma et al. Therefore, many differences existed between the present model and previous models, but there are several benefits of using a canine model rather than smaller animal models, such as rat or mouse models. First, the common bile duct and pancreatic duct in dogs were separated. Collection of the pure PF required only pancreatic duct cannulation. The simpler operative procedure perhaps resulted in a decreased alteration of pancreatic exocrine function. Some of the above experiments altered the digestive tract to collect the pure PF. Such alteration can affect the exocrine function of the pancreas. Second, our present observations of pancreatic secretion were made in conscious and fasting dogs, which could exclude the effect of anesthesia and food. Third, dogs are bigger animals and have a larger volume of PF than rats. The greater the volume of PF, the more accurate the results are. Fourth, because of the good nutrition support, our model was suitable for performing long and dynamic observations. There is a secretory cycle of the pancreas, so a long observation period decreases the error. The control of pancreatic secretion is complex and highly regulated by neural and hormonal factors [16,17]. An ideal model for the research of pancreatic exocrine function requires less interference with the pancreatic exocrine regulating mechanisms. For the above reasons, our animal model seems more reliable and suitable for the investigation of pancreatic exocrine function and the underlying mechanisms of pancreatic exocrine function in AP.

The physiological importance of both the acinar and ductal cells is well known. Pancreatic acinar cells synthesize, store, and secrete digestive enzymes, and the duct cells secrete a bicarbonate-rich isotonic solution that is necessary for the transportation of digestive enzymes into the duodenum and neutralization of gastric acid [18,19]. The sorting of these enzymes depends on the pH-dependent aggregation of the proteins in the trans-Golgi network, which result in immature granules in AP [18]. Premature activation of digestive zymogens within the pancreatic acinar cells is a key step in initiating AP [4]. Our results showed that the PF volume, amylase and lipase activities, and protein and bicarbonate concentrations in PF were decreased in our AP canine model. This result suggests that the digestive enzyme synthesis and ductal cell secretion in acinar cells are impaired.

The correlation between pancreatic exocrine function and ultrastructural changes in the pancreas has been under-explored. There are several possible explanations for the ultrastructural changes responsible for the decreased pancreatic exocrine function in dogs after AP. Previous studies suggested the possibility that the dysfunction of cellular organelles may lead to the development of AP [20–22] and decrease pancreatic exocrine function [23]. For normal secretion, the integrity of the pancreatic acinar and duct cells is mandatory. During pancreatitis, the responsiveness and sensitivity of pancreatic cells are markedly reduced [11]. Damage of pancreatic cells caused by AP was confirmed by electron microscopy in our study. Electron microscopy revealed necrosis in individual acinar cells, dilatation and degranulation in most of the RER, and the dropping off of some of the ribosomes from the RER. The mitochondria were swollen and contained short and broken cristae. Large autophagic vacuoles were seen.

Ribosomes are essential for the synthesis of protein. RER degranulation will reduce the synthesis of proteins. Mitochondria generate most of the ATP required for cell function in normal pancreatic cellular physiology [24]. Recent findings indicated that mitochondrial damage and ATP depletion played a central role in the development of AP [10,20,22,24], which caused cellular injury to both the pancreatic acinar and ductal cells. Autophagy is a lysosomal degradation pathway that is essential for survival, differentiation, development, and homeostasis [25]. The inefficient lysosomal degradation in pancreatitis results in retarded autophagic flux. In AP, the autophagy process of the acinar cells is dysfunctional and no longer beneficial to the cell but toxic, leading to tissue damage [26]. In summary, the mitochondrial injury to both the pancreatic acinar and ductal cells, the RER dilatation and degranulation in the pancreatic acinar cells, and the retardation of autophagic vacuoles in the pancreatic acinar cells in the present study suggested the significance of the impaired enzyme synthesis and secretion in the canine model. Therefore, the most likely explanation for reduced pancreatic exocrine is that damage to acinar and ductal cells, after the onset of AP. Other possible explanations for pancreatic hyposecretion may also involve an increased release of inflammatory mediators, disturbances in pancreatic microcirculation, and dysfunction of intracellular calcium homeostasis.

In conclusion, we characterized a novel canine model of AP that is suitable for the research of pancreatic exocrine function. The present study demonstrated persistent PF hyposecretion and decreased enzyme and bicarbonate secretion during the early stage after the onset of AP in dogs. Alterations were detected by electron microscopy, such as swelling of mitochondria, acinar cell necrosis, RER dilatation and degranulation, and retardation of autophagic vacuoles, that may, at least in part, account for pancreatic exocrine hypofunction in AP.

None of the current animal models are ideally compatible with the clinical scenarios of pancreatitis. However, the present canine model may be useful for the assessment of novel therapeutic measures in AP.

## Supporting Information

S1 Data The PF volume, outputs of amylase activity, lipase activity, protein concentration, bicarbonate concentration and pH value in the PF in a canine AP model. Serum amylase and lipase activities in a canine AP model. The PF volume in a canine AP model (Fig A). Output of amylase activity in the PF in a canine AP model (Fig B). Output of lipase activity in the PF in a canine AP model (Fig C). Output of protein concentration in the PF in a canine AP model (Fig D). Bicarbonate concentration and pH value in the PF in a canine AP model (Fig E). Serum amylase and lipase activities (Fig F).
(RAR)

S1 Fig Histopathological examination of the canine pancreas. A pancreas from the control group (×100) (Fig A). A pancreas from the control group (×200) (Fig B). A pancreas from the control group (×400) (Fig C). A pancreas from the AP group (×100) (Fig D). A pancreas from the AP group (×200) (Fig E). A pancreas from the AP group (×400) (Fig F).
(RAR)

S2 Fig Electron micrographs of the canine pancreas from the control group. Electron micrograph of pancreatic acinar cells from the control group (scale bar = 1 μm) (Fig A). Electron micrograph of normal pancreatic acinar cells from the control group (scale bar = 2 μm) (Fig B). Electron micrograph of rough endoplasmic reticulum (RER) from the control group (scale bar = 0.5 μm) (Fig C). Electron micrograph of mitochondria (Mi) from the control group (scale bar = 0.2 μm) (Fig D).
(RAR)

S3 Fig Electron micrographs of the canine pancreas from the AP group. Electron micrograph of pancreatic acinar cells from the AP group (scale bar = 1 μm) (Fig A). Electron micrograph of pancreatic acinar cells from the AP group (scale bar = 2 μm) (Fig B). Electron micrograph of RER from the sodium taurocholate-induced AP group (scale bar = 0.5 μm) (Fig C). Electron micrograph of mitochondria (Mi) from the sodium taurocholate-induced AP group (scale bar = 0.2 μm) (Fig D). (E) Electron micrograph of autophagic vacuoles from the AP group (scale bar = 1 μm) (Fig E). Electron micrograph of acinar cell necrosis from the AP group (scale bar = 1 μm) (Fig F).
(RAR)
